# How Body Orientation Affects Concepts of Space, Time and Valence: Functional Relevance of Integrating Sensorimotor Experiences during Word Processing

**DOI:** 10.1371/journal.pone.0165795

**Published:** 2016-11-03

**Authors:** Martin Lachmair, Susana Ruiz Fernandez, Nils-Alexander Bury, Peter Gerjets, Martin H. Fischer, Otmar L. Bock

**Affiliations:** 1 Leibniz-Knowledge Media Research Center, Tuebingen, Germany; 2 Institute of Physiology und Anatomy, German Sport University Cologne, Cologne, Germany; 3 Department of Psychology, University of Potsdam, Potsdam, Germany; Universiteit van Amsterdam, NETHERLANDS

## Abstract

The aim of the present study was to test the functional relevance of the spatial concepts UP or DOWN for words that use these concepts either literally (space) or metaphorically (time, valence). A functional relevance would imply a symmetrical relationship between the spatial concepts and words related to these concepts, showing that processing words activate the related spatial concepts on one hand, but also that an activation of the concepts will ease the retrieval of a related word on the other. For the latter, the rotation angle of participant’s body position was manipulated either to an upright or a head-down tilted body position to activate the related spatial concept. Afterwards participants produced in a within-subject design previously memorized words of the concepts space, time and valence according to the pace of a metronome. All words were related either to the spatial concept UP or DOWN. The results including Bayesian analyses show (1) a significant interaction between body position and words using the concepts UP and DOWN literally, (2) a marginal significant interaction between body position and temporal words and (3) no effect between body position and valence words. However, post-hoc analyses suggest no difference between experiments. Thus, the authors concluded that integrating sensorimotor experiences is indeed of functional relevance for all three concepts of space, time and valence. However, the strength of this functional relevance depends on how close words are linked to mental concepts representing vertical space.

## Introduction

The grounded view of language processing postulates that semantic representations in the human brain are obligatorily linked to sensorimotor representations [1]. When interacting with the world people encounter words together with the related objects, states, or events that are experienced according to the general physical laws on Earth. As a result words become associated with sensorimotor representations reflecting interactive experiences within our environment. When these same words are processed again at a later time, the related sensorimotor representations are activated in a simulation-like process to create meaning [2]. Evidence for the reactivation of such rich representations comes for example from a TMS-study which demonstrates specific functional links between action and language systems during lexical processing [3].

### Literal use of vertical spatial meaning

This associative mechanism provides a plausible explanation for mental representations of words that refer to concrete entities. For example a large amount of empirical findings support this view for words that integrate vertical spatial meaning attributes in a *literal* way, be they verbs like “rise” or “fall” [4] or nouns like “sun” or “ground” [5] or adjectives like “up” or “down” [6]. For example, understanding a word like “cloud” includes remembering the perceptual experience of the typical location above the observer, thereby activating specific sensorimotor attributes of the referent. This information is stored in the brain and becomes automatically reactivated when processing the word “cloud”, obligatorily shifting our attention upwards [7]. The grounding of spatial linguistic labels in spatial experience has also been shown in a study by Ansorge et al. [6]. There, the authors presented participants with prime-target word pairs consisting of adjectives that denote a position or a direction in vertical space (e.g., “oben/on top”, “hinauf/upward” vs. “unten/down”, “hinab/downward”). Participants had to perform a manual response upwards or downwards, according to the target word. The results of their study showed that the response was affected by the prime although presented subliminally, showing faster responses in congruent compared to incongruent conditions. The authors argue in line with the view of grounded cognition, interpreting their findings as the result of an automatic reactivation of experience-based spatial representations while processing the associated spatial word.

### Metaphorical use of vertical spatial meaning

Interestingly, the relationship between language processing and spatial dimensions of responses has also been shown for rather abstract mental concepts that integrate spatial meaning attributes in a *metaphorical* way. For example in a study by Meier and Robinson [8] with words associated to the abstract concept of valence, they showed that words like “pride” denoting positive valence are associated with an upper position in space whereas words like “sadness” denoting negative valence are associated with a lower position in space.

Other empirical findings suggest that temporal concepts are represented according to a mental time line, representing the past behind or to the left and the future ahead or to the right [9–14]. Interestingly, there is also evidence that temporal representations integrate meaning attributes representing vertical space. For example a recent study by Ruiz Fernandéz and colleagues [15] showed that temporal words related to the future facilitate responses to a target located in the upper space compared to a target located in lower space. The reverse pattern applied to words related to the past; responses to a subsequent target located in lower space were facilitated compared to a subsequent target located in the upper space. In the domain of language processing such findings are typically explained with the metaphorical mapping hypothesis [16]. Accordingly, the meaning of a word related to an abstract concept like valence or time is represented in terms of meaning attributes that are associated with sensorimotor representations of more concrete meaning dimensions like space.

### Functional relevance of experiential representations

Interestingly, by far the largest part of experimental studies have in common that they employ experimental paradigms that only allow conclusions of a causal relationship in one direction, showing an influence of word processing on *subsequent* sensorimotor processes. Therefore, it is not clear if reactivating spatial representations as part of language processing is of functional relevance for comprehension or a mere by-product. Indeed, this is a central criticism against a grounded view of language processing [17]. Thus, if a re-activation of representations that integrate attributes related to vertical space is of functional relevance for word processing, we would not only expect that word processing affects subsequent sensorimotor processes. We should also find strong performance biases in the opposite direction, namely that sensorimotor processes affect subsequent word processing. Therefore, it is relevant to find out to what degree these spatial associations are experience-specific in nature and if they are therefore functional relevant for both literal *and* metaphorical use of spatial meaning dimensions in the representation of related concepts. A functional relevance would imply a symmetrical relationship between spatial representations and word representations. For words using spatial representations literally, there is evidence of early and automatic activation of spatial representations (see above). Therefore, a very close link between spatial representations and words that use vertical spatial representations literally is assumed, suggesting a symmetrical relationship. With this regard the study by Meier and Robinson [8] suggests an asymmetrical relationship between spatial representations and valence word processing. In Experiment 3 of their study, participants were presented with a probe at the top or bottom of the computer screen. The task was to determine the location of the probe by saying “up” or “down”. This should activate a representation of the concepts UP or DOWN. Subsequently, participants had to discriminate between positive or negative words by pressing an upper or lower key according to valence. The results of this experiment showed no effect of the activation of the concept UP or DOWN on the subsequent key responses. Together with the other results of the study this suggests an activation of literal meaning when metaphorical meaning is generated but no activation of metaphorical meaning when literal meaning is generated. However, this seems to be in contrast to other studies that found an effect of body postures (including facial expressions) on the retrieval of positive and negative life experiences [e.g., 18]. A possible explanation could be that participants employ a different strategy for rather complex material. But still, this observation casts some doubt over the strong claim of asymmetry for the concept of valence.

Interestingly, a similar picture can be painted for the abstract concept of time. There is evidence showing an asymmetrical relationship between spatial and temporal representations. For example, people are unable to ignore irrelevant spatial information when making judgments about duration, but not the converse [19]. A follow-up study with children pointed to the same asymmetry [20]. However, these studies investigated time as duration during motor action or together with distance estimation which are rather dynamic processes. Moreover, there is evidence that temporal and spatial representations overlap in parietal brain regions, particularly in the intraparietal sulcus, representing magnitude (e.g. [21]; cf. [22]). This suggests a much closer link between spatial and temporal representations than between spatial and valence-related representations. Thus, it is not clear if the spatial mapping of abstract temporal concepts like future or past expressed by related words would show a similar asymmetrical relationship (cf. [23]).

**Fig 1:**
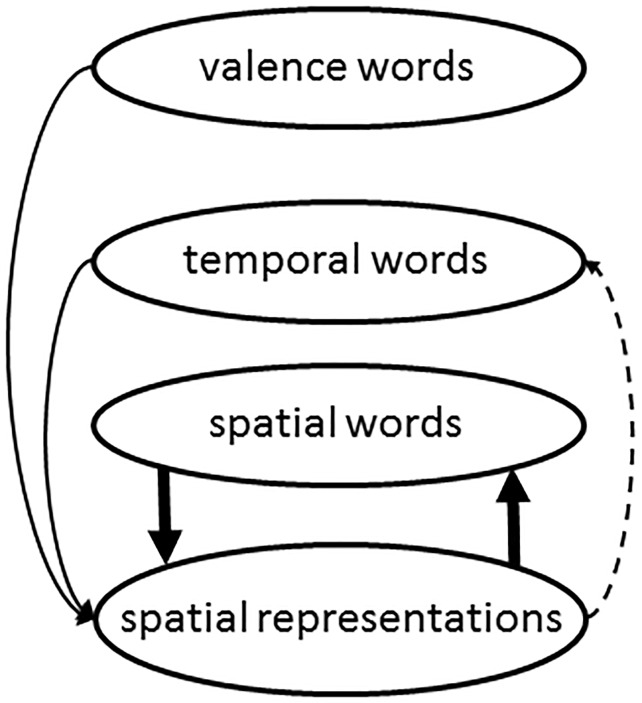
Graphical overview. This overview illustrates the relevance of sensorimotor experiences for the concepts space, time and valence. Arrows in both directions suggest a functional relevance.

Based on these theoretical considerations and the available empirical evidence, we derived a graphical overview that illustrates the functional relevance of integrating spatial meaning attributes in representations of words using vertical spatial meaning either literally (spatial words) or metaphorically (temporal and valence words), which is illustrated in Fig 1. Accordingly, the graphical overview illustrates a symmetric relation between spatial words and spatial representations, suggesting a functional relevance of spatial representations for words using them literally. The dotted arrow reflects the fact that there is evidence for a closer link between temporal and spatial representations according to a common representational system of time and space [21]. Additionally, it considers evidence for an asymmetrical relationship between spatial representations and temporal words, which would speak against a functional relevance of spatial representations for temporal words. The graphical overview also assumes that valence representations activate spatial representations, but not the converse. This is tantamount with an asymmetric relationship between spatial representations and valence words, suggesting no functional relevance.

**Fig 2:**
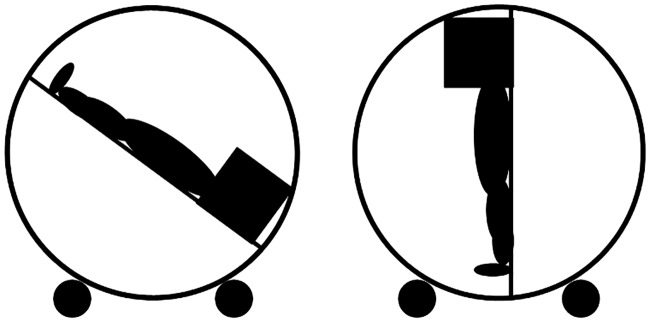
The experimental setup. The two body positions head-down tilt and upright.

The aim of the present study was to find evidence for hypotheses derived from this graphical overview in Fig 1 that illustrates the relationship of the functional relevance of integrating spatial representations of the three concepts space, time and valence. Therefore, we investigated how the processing of words that integrate vertical spatial meaning literally or metaphorically is affected when sensorimotor experience is manipulated. This was achieved by manipulating the rotation angle of a person in supine position. The assumption is that the mental representations of the spatial concepts of UP and DOWN are activated due to the immediate sensorimotor experience according to the rotation angle. For this purpose a gym wheel with an installed stretcher was used (Fig 2). Participants lying on this stretcher were tested in two different body positions (upright and head-down tilted) under conditions of uncertain orientation without information from vision, from their hands or from their feet (cf. Fig 2). The only information that allowed the determination of one’s body orientation stemmed from tactile cues of the skin, the back (through the contact with the stretcher) and the information provided by the vestibular system. During experiencing these body positions, a word-recall task was conducted; participants should randomly recall beforehand memorized words which are related to the concepts UP or DOWN either literally (Experiments 1 with spatial adjectives) or metaphorically (Experiment 2 with temporal and Experiment 3 with valence related adjectives). Please note that participants executed all three experiments in a within-subject design. This allowed a direct comparison of the influence of the sensorimotor experience on the processing of the different concepts.

Based on the proposed graphical overview in Fig 1, the hypothesis for Experiment 1 (spatial words) is that due to an easier activation of the concept UP in upright body position the proportion of words related to that concept (UP-words) would be larger than for words related to the concept DOWN (DOWN-words). The reversed should apply for head-down tilted position due to an easier activation of the concept DOWN. There, it was expected that the proportion of DOWN-words would be larger compared to that of UP-words. For Experiment 2 (temporal words), it was expected that the influence of body position on the proportion of UP- and DOWN-words would be reduced compared to that of literal words in Experiment 1. But still, due to a common sense of magnitude we expected a similar proportion-pattern as in Experiment 1. For Experiment 3, it was expected that the manipulation of body position has no influence on the proportion of the words, in line with the findings of Meier and Robinson [8].

## Experiment 1

### Participants

Twenty four German native speakers (12 female; *M*_*age*_ = 22.1 years, *sd = 2*.*9*) took part in this experiment. All were students of the German Sports University in Cologne. They received a financial reimbursement of 8 Euros per hour. The study protocol was approved in advance by the Ethics Commission of the German Sport University of Cologne. Each subject provided written informed consent before participating.

## Material

For the Word-Recall Task a wordlist of eight German adjectives was employed (cf. [6]). Four of them denoted a position up in vertical space and four a position down: *oben*/*up*, *unten*/*down*, *hinauf*/*uphill*, *hinab*/*downhill*, *hoch*/*high*, *tief*/*low*, *rauf*/*upwards* and *runter*/*downwards*. The adjectives were controlled for frequency with the "Wortschatz Portal" of the University of Leipzig, showing no differences between UP- and DOWN-adjectives (*t*(6) = 0.60, *p* > .58).

### Experimental setup

The participants rested on a stretcher, installed on a standard gym wheel with a diameter of 220 cm. The gym wheel was placed in a darkened room on castors to adjust the participants’ body positions into head-down tilted and upright (cf. Fig 2). Note that in every position neither participants’ feet nor their hands had tactile input that could give information about the spatial orientation of the participant. Further, participant’s head was surrounded with an opaque black box with an edge length of 40 cm and opaque black curtains to the chest side. Thus, they had also no visual input from the surrounding. The participants were covered with a rubber foam mat, fixed on the stretcher with four hook-and-loop tapes placed right under the knees, thighs, over the hips and over the chest (cf. Fig 2). Oral responses were recorded with two voice recorders.

### Procedure and Design

First, the participants learned to memorize the eight words of the wordlist to perfection, which took less than 5 minutes. Afterwards they were fixed on the stretcher with the rubber foam mat and the hook-and-loop tapes on the gym wheel. This procedure took about 10 minutes. After closing the opaque curtains of the box surrounding the head, participants were confused about their exact body position with random gym wheel turnings clockwise and counter-clockwise. The body positions were tested block-wise: block “upright” and block “head-down tilted”. The order of these two blocks was counterbalanced across participants. Within each block participants were instructed to randomly recall and produce one of the previously memorized words under time pressure according to ticks of a digital metronome (0.25 Hz, 40 ticks) which was presented via headphones. This procedure was intended to keep participants from thinking about recall strategies (cf. [24, 25]). Thus, we obtained from every participant performance of 40 trials (each trial is a randomly recalled and produced word out of the eight memorized words) in each of the two body positions resulting in 80 experimental trials of each participant. The design was a 2 (Word Category UP vs DOWN) x 2 (Body Position upright vs. head-down tilted) x 2 (Order of Body Position) design with repeated measurement, with Word Category and Body Position as within-participant factors and with Order of Body Position as between-participants factor. The dependent variable was measured as the proportion of recalled UP- and DOWN-words in each body position.

### Results and Discussion

**Fig 3:**
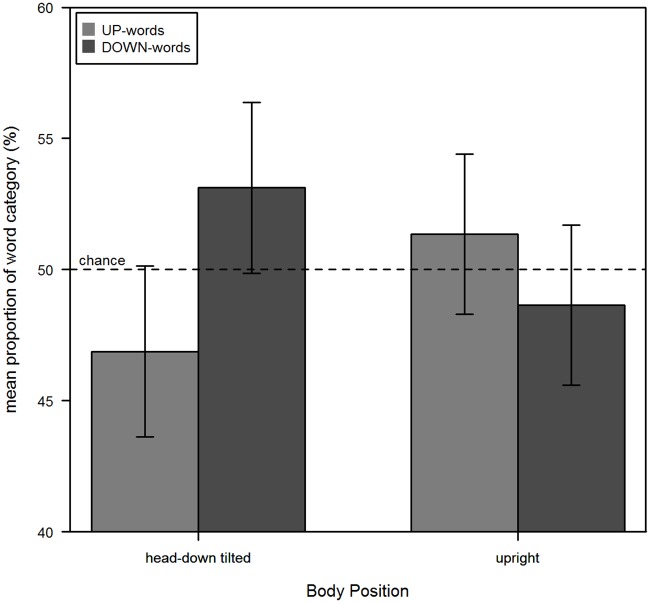
Results of Experiment 1. The mean proportion of UP- and DOWN-words of head-down tilted and upright body position. Error bars represent the 95% confidence interval for within subject designs [28].

Participants remembered and produced all previously learned words. The amount of UP- and DOWN-words recalled in each body position is illustrated in Fig 3. The chance level of Word Category (UP vs. DOWN) for word recall was 50%. To test the hypothesis of the present study, the data for the upright and the head-down tilted position were analyzed with an analysis of variance (ANOVA) for repeated measurement. Keeping in mind controversies regarding confirmation of null hypothesis using traditional statistical inference we also determined JZS Bayes factors with default prior scales as described in [26,27]. These results are summarized in Table 1. As illustrated, main effects of Body Position, Order of Body Position and Word Category were not significant, as well as all interactions with Order of Body Position. As expected, we found a significant interaction between Body Position and Word Category with a higher amount of up-words recalled in the upright position and a lower amount of up-words in the head-down tilted position. Further post-hoc analyses showed that the difference between UP-words and DOWN-words in upright position was not significant (*t*(23) = 1.08, *p* = .29, *SS* = 88, SS_res_ = 1750, BF_10_ = 0.73), but significant in head-down tilted position (*t*(23) = -2.32, *p* = .03, *SS* = 469, SS_res_ = 2006.2, BF_10_ = 17.88). Thus, this result can be interpreted as reflecting a congruency between sensorimotor experience provided by body orientation and meaning representations of spatial words. To exclude an influence of an early word recall on the recall of the same word later in time, additional post-hoc analyses were conducted to explore the distribution of the word categories for each body position over the time of the experimental task. Therefore, we split the data for each body position into two halves and determined the frequencies of each word category. With the resulting 2x2 table a chi-square test was conducted that showed no significant difference between the frequencies of each word category in each half for body position “upright” (*χ*^*2*^(1) = 0.02, *p* = 0.90, BF_10_ = 0.18), as well as for body position “head-down tilt” (*χ*^*2*^(1) = 0.71, *p* = 0.40, BF_10_ = 0.12). Thus, it can be concluded that early recalls of words had no influence on the counting of later recalls. This supports the proposed interpretation of the results and strengthens the view of a functional relevance of spatial sensorimotor information in mental representations of words that integrate vertical spatial attributes literally.

**Table 1:** ANOVA-table and Bayes Factors of Experiment 1.

	Df	SS	F	p	BF_10_
Error: Subject					
Order Body Pos.	1	0	0.62	0.44	0.26
Residuals	22	0			
Error: Subject:Body Position					
Body Position	1	0	0.35	0.56	0.21
Body Position: Order Body Pos.	1	0	0.93	0.35	0.02
Residuals	22	0			
Error: Subject: Word Category					
Word Category	1	75.3	0.88	0.36	0.44
Word Category: Order Body Pos.	1	2.3	0.03	0.87	0.88
Residuals	22	1891.1			
Error: Subject:Body Pos.:Word Cat.					
Body Position:Word Category	1	481.5	5.71	0.03	3.45
Body Pos.:Word Cat.:Order Body Pos.	1	6.5	0.08	0.78	0.03
Residuals	22	1855.7			

## Experiment 2

The previous experiment showed a significant influence of body position on the availability of words denoting a location up or down in vertical space literally. Thus, the objective of this experiment was to find out to what extend sensorimotor cues stemming from different body positions in space are strong enough to even affect the availability of temporal concepts referring to future and past, which are merely metaphorically related to vertical spatial meaning.

### Participants

The same twenty four German native speakers (12 female; Mage = 22.1 years, sd = 2.9) as in Experiment 1 took part in this experiment. They were tested at a second session of at least two days apart. They received another financial reimbursement of 8 Euros per hour. The study protocol was approved in advance by the Ethics Commission of the German Sport University of Cologne. Each subject provided written informed consent before participating.

### Material

Four past and four future adjectives were used: “morgen”/tomorrow, “übermorgen”/the day after tomorrow, “gestern”/yesterday, vorgestern”/the day before yesterday, “vorhin”/a little while ago, “damals”/at that time, “künftig”/in the future, “demnächst”/soon. The adjectives were again controlled for frequency with the "Wortschatz Portal" of the University of Leipzig, showing no differences, *t*(6) = .13, *p* > .90.

### Experimental setup

The same paradigm was used as in Experiment 1.

### Procedure and design

The same procedure was used as in Experiment 1.

### Results and Discussion

**Fig 4:**
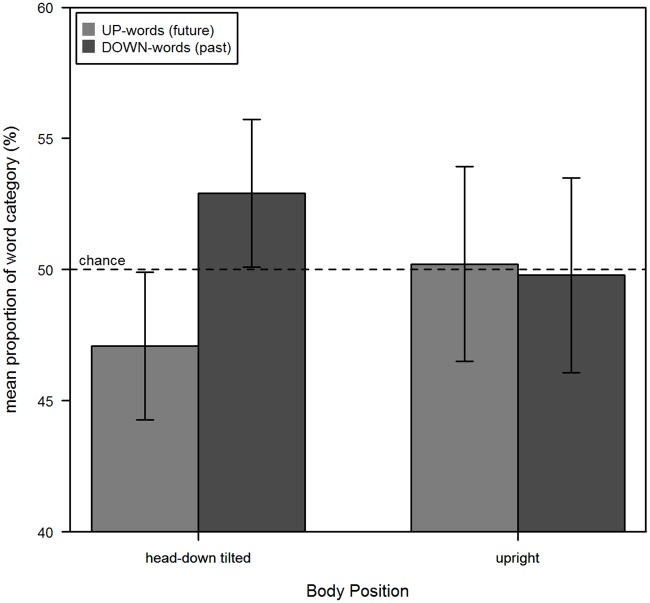
Results of Experiment 2. The mean proportion of future and past words recalled in each body position. Error bars represent the 95% confidence interval for within subject designs [28].

Participants remembered and produced all previously learned words. The amount of UP- and DOWN-words recalled in each body position is illustrated in Fig 4. As in Experiment 1, the data for the upright and the head-down tilted position were analyzed with an analysis of variance (ANOVA) for repeated measurement. Together with their Bayes factors these results are summarized in Table 2. Accordingly, main effects of Body Positionand Word Category were not significant, but not the order of body position. The interaction between Body Position and Order of Body Position was marginal significant. The interactions between Word Category and Order of Body Position and crucially the three-way interaction between Word Category, Body Position and the order of Body Position were not significant. The interaction between Body Position and Word Category was marginally significant, although the Bayes factor provided no evidence for this interaction. Further post-hoc analyses showed that the difference between UP- and DOWN-words in head-down tilted position was significant (*t*(23) = -2.51, *p* = .019, *SS* = 408.3, SS_res_ = 1491.7, BF_10_ = 33.68), showing more DOWN-words (52.92%) than UP-words (47.08%). However, in upright position this difference was not significant (UP-words: 50.21%, DOWN- words: 49.79%; *t*(23) = .14, *p* = .89, *SS* = 2.1, SS_res_ = 2597.9, BF_10_ = 0.28). Thus, although there is a tendency comparable to the results of Experiment 1, there was no significant interaction between body position and the availability of temporal concepts. As in Experiment 1, we split the data for each body position into two halves and determined again the frequencies of each word category. A chi-square test showed no significant difference between the frequencies of each word category in each half for body position “upright” (*χ*^*2*^(1) = 0.38, *p* = 0.56, BF_10_ = 0.14), as well as for body position “head-down tilt” (*χ*^*2*^(1) = 0.0, *p* = 1, BF_10_ = 0.12).

**Table 2:** ANOVA-table and Bayes Factors of Experiment 2.

	Df	SS	F	p	BF_10_
Error: Subject					
Order Body Pos.	1	0	5.73	0.03	0.26
Residuals	22	0			
Error: Subject:Body Position					
Body Position	1	0	0.75	0.40	0.29
Body Position: Order Body Pos.	1	0	3.11	0.09	0.02
Residuals	22	0			
Error: Subject: Word Category					
Word Category	1	176.0	1.60	0.22	1.13
Word Category: Order Body Pos.	1	4.2	0.04	0.85	0.08
Residuals	22	2419.8			
Error: Subject:Body Pos.:Word Cat.					
Body Position:Word Category	1	234.4	3.10	0.09	0.60
Body Pos.:Word Cat.:Order Body Pos.	1	0.0	0.0	0.0	1.00
Residuals	22	1665.6			

Taken the results of Experiment 1 into account this indicates a rather low functional relevance of integrating sensorimotor experience to the meaning of temporal words.

## Experiment 3

As described above a metaphorical relationship between language processing and vertical space has also been shown for words denoting valence. Meier and Robinson showed that words denoting positive valence are associated with an upper position in space whereas words denoting negative valence are associated with a lower position in space. However they also showed an asymmetrical relationship between valence and spatial representations [8]. Thus, as illustrated in the graphical overview in Fig 1, a comparatively weak effect, if any, of spatial attributes on processing words denoting valence was expected.

### Participants

Participants were the same as in Experiment 1. However, they were tested in a third session of at least another two days apart. Participants received another financial reimbursement of 8 Euros per hour. The study protocol was approved in advance by the Ethics Commission of the German Sport University of Cologne. Each subject provided written informed consent before participating.

### Material

Four positive and four negative adjectives were used: “*gefühlvoll*”/*lyrical*, “*zärtlich*”/*tender*, “*phantastisch*”/*amazing*, *vergnügt*”/*amused*, “*dämlich*”/*goony*, “*unangenehm*”/*awkward*, “*hässlich*”/*ugly*, “*verwahrlost*”/*bedraggled*. The adjectives were again controlled for frequency with the "Wortschatz Portal" of the University of Leipzig, showing no differences, *t*(6) = .51, *p* > .63.

### Experimental Setup

The same paradigm was used as in Experiment 1.

### Procedure and Design

The same procedure was used as in Experiment 1.

### Results and Discussion

**Fig 5:**
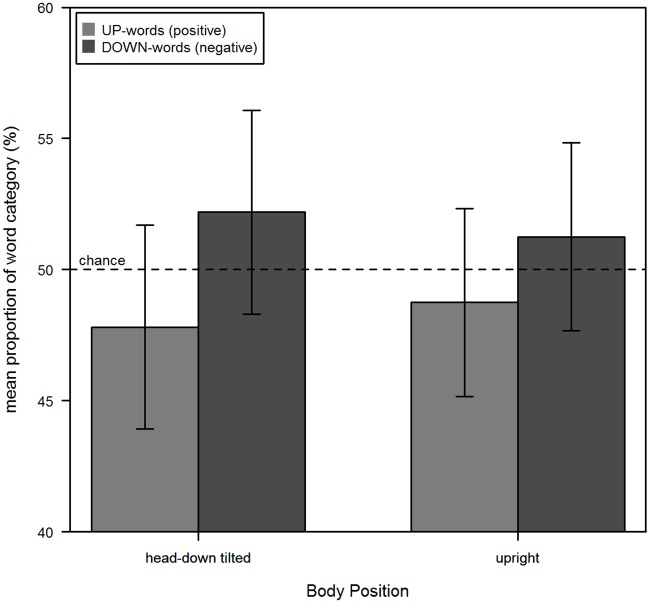
Results of Experiment 3. The mean proportion of positive and negative words recalled in each body position. Error bars represent the 95% confidence interval for within subject designs [28].

Participants remembered and produced all previously learned words. The amount of UP- and DOWN-words recalled in each body position is illustrated in Fig 5. As in the other experiments, the data for the upright and the head-down tilted position were analyzed with an analysis of variance (ANOVA) for repeated measurement. Together with their Bayes factors these results are summarized in Table 3. Accordingly, Main effects of Body Position, Word Category and Order of Body Position were not significant. Interestingly, the interaction between Body Position and Word Category was also not significant, as well as the three-way interaction with Order of Body Position. This result supports the view of an asymmetrical relationship between representations of valence and spatial representations [8]. A chi-square test as in Experiment 1 and 2 showed no significant difference between the frequencies of each word category in each half for body position “upright” (*χ*^*2*^(1) = 0.10, *p* = 0.75, BF_10_ = 0.13), as well as for body position “head-down tilt” (*χ*^*2*^(1) = 0.07, *p* = 0.80, BF_10_ = 0.13).

**Table 3:** ANOVA-table and Bayes Factors of Experiment 3.

	Df	SS	F	p	BF_10_
Error: Subject					
Order Body Pos.	1	0	0.02	0.90	0.26
Residuals	22	0			
Error: Subject:Body Position					
Body Position	1	0	0.60	0.45	0.21
Body Position: Order Body Pos.	1	0	0.90	0.35	0.01
Residuals	22	0			
Error: Subject: Word Category					
Word Category	1	284.0	1.97	0.17	2.14
Word Category: Order Body Pos.	1	21.0	0.15	0.71	0.04
Residuals	22	3164			
Error: Subject:Body Pos.:Word Cat.					
Body Position:Word Category	1	21.1	0.26	0.62	0.15
Body Pos.:Word Cat.:Order Body Pos.	1	0.3	0.0	0.96	0.0
Residuals	22	1822.4			

### Comparing space, time and valence

So far, the results of the analyses of the three experiments suggest on one hand a closer relationship between processing spatial words and temporal words. On the other hand, they suggest a looser relationship between processing spatial words and valence words or between temporal words and valence words. However this is derived from analyses that do not compare the experiments directly. Thus, introducing the within-participant factor Experiment with three levels we conducted another post-hoc ANOVA to shed more light on this issue. These results are illustrated in Table 4. Against the background of the result patterns of Experiment 1, 2 and 3 it was expected that the three-way interaction between Word Category, Body Position and Experiment would not reach significance due to quite similar result patterns of Experiment 1 and 2 and due to similar effects of the head-down tilted body position across all three experiments. Moreover the two-way interaction between Word Category and Body Position should be at least reduced due to the opposing result-pattern of Experiment 3. Indeed, this is what the ANOVA showed. The three-way interaction was not significant and the two-way interaction only marginal significant. The lack of a significant two-way interaction could be interpreted as support for a graded relationship between sensorimotor experiences and the concepts of space, time and valence although the three-way interaction is not significant. However, this picture is not supported by the related Bayes factors. The interpretation of these Bayes factors is that there is no difference between the experiments. Thus, the conclusion from that would be that sensorimotor experiences are indeed of functional relevance for all three mental concepts of space, time and valence.

**Table 4:** ANOVA-table and Bayes Factors of analysis between experiments.

	Df	SS	F	p	BF_10_
Error: Subject					
Residuals	23	0			
Error: Subject:Body Position					
Body Position	1	0	1.53	0.23	0.13
Residuals	23	0			
Error: Subject: Word Category					
Word Category	1	501.0	3.32	0.08	18.06
Residuals	23	3474			
Error: Subject: Experiment					
Experiment	2	0	0.69	0.51	0.04
Residuals	46	0			
Error: Subject:Body Pos.:Word Cat.					
Body Position:Word Category	1	584	3.69	0.07	134.73
Residuals	23	3641			
Error: Subject:Body Pos.:Experiment					
Body Position:Experiment	2	0	0.73	0.49	0.0
Residuals	46	0			
Error: Subject:Word Cat.:Experiment					
Word Category:Experiment	2	34	0.19	0.83	0.06
Residuals	46	4029			
Error: Subject:Body Pos.:Word Cat.:Exp					
Body Position:Word Category:Exp	2	153.3	2.06	0.14	0.01
Residuals	46	1709.2			

## General Discussion

The aim of the present study was to investigate the functional relevance of integrating spatial attributes into meaning representations of words that use these spatial attributes either literally or metaphorically (see Fig 1). This was examined by means of a within-subjects design. From a graphical overview representing results of the relevant research so far, we derived hypotheses that were tested by investigating the influence of body position (upright and head-down tilt) on the availability of spatially related concepts. Concretely, we tested if the perception of body position has an influence on the retrieval of words that integrate spatial meaning attributes either literally or metaphorically. We focused on ease of word retrieval as it is well known that comprehension of linguistic expressions relies on such retrieval processes (e.g., [29]). Thus, the findings of the present study can be considered relevant for linguistic processing in general. The results of Experiment 1 show that the availability of spatial concepts expressed by words related literally to spatial meaning is indeed significantly affected by body position. This is supported by Bayesian analysis. An upright position promoted retrieval of words denoting a location up in space whereas a head-down tilted position promoted retrieval of words denoting a location down in space. The grounded cognition approach provides a plausible explanation for this finding. The basic “perceptual mechanisms” of the human brain have been shaped by its physical environment over thousands of years. Determining one’s position in the environment is of course of utmost importance for survival. Thus, it is conceivable that information on body orientation is strongly integrated into higher cognitive processes, which include the representation and availability of spatial language. This is supported by studies suggesting an automatic activation of spatial information, when words are processed that integrate spatial meaning attributes literally (e.g., [6, 7]). Beyond that, the results of Experiment 1 even show the reverse relationship: the preceding perception of spatial information provided by body orientation affects the availability of words related to that spatial information. This suggests a symmetrical relationship between representations of spatial language and the representation of spatial body states in that not only word processing affects subsequent spatial responses (e.g. [6, 7]), but also spatial body perception affects subsequent retrieval of spatial words. This is reflected in the symmetrical relationship between spatial words and spatial representations as illustrated in the graphical overview in Fig 1.

For temporal words this overview provides two possible relationships. The first supports the view of an asymmetrical relationship with spatial representations in line with the metaphorical mapping hypothesis as suggested by Casasanto et al. [19]. The second supports the view of a common representational platform for time and space suggesting a symmetrical relationship and thus a functional relevance of spatial representations. The results of an ANOVA showed a marginally significant interaction between body position and temporal word categories. It is striking that the upright body position shows no effect on word proportions, neither for Experiment 1 nor for Experiment 2, which is also reflected in the related Bayes factors. A plausible explanation could be that this position is a canonical body position. In contrast, a head-down tilted body position is rather unusual and could therefore have a stronger impact on word proportions. Thus, the ANOVA of Experiment 2 suggest on the one hand that temporal words are less sensitive to body position compared to words related to literal spatial meaning (Experiment 1). On the other hand these results also suggest that temporal concepts are more sensitive to the employed body positions with regard to spatial dimensions than concepts of positive and negative valence as examined in Experiment 3.

However, although the individual analyses of the experiments suggest a graded relationship between concepts that integrate sensorimotor experiences literally and concepts that integrate sensorimotor experiences metaphorically, the results of comparing the experiments directly, in particular the high Bayes factor of the two-way interaction between body position and word category and the very low Bayes factor of the three-way interaction including Experiment as factor, rather showed that there is no difference between these concepts with regard to the integration of sensorimotor experiences stemming from different body positions. This suggests a functional relevance of sensorimotor experiences for all three concepts of space, time and valence.

What does this mean for a grounded view of word processing? The presented results support the view of a functional relevance of integrating spatial experiences for both words that use vertical spatial meaning literally or metaphorically. However, the results also suggest that the strength of functional relevance depends on how close experiential spatial representations are linked to mental concepts representing vertical space.
